# Examining the relationship between temperature and firearm violence: A comparison of metrics

**DOI:** 10.1371/journal.pone.0358196

**Published:** 2026-09-30

**Authors:** Hao Zhang, Bryce Hruska

**Affiliations:** Department of Public Health, Syracuse University, Syracuse New York, United States of America; Sustainable Psychological Science, AUSTRALIA

## Abstract

**Objective:**

To examine and compare the predictive capacities of four temperature metrics on firearm violence: daily maximum temperature, daily apparent maximum temperature, daily deviation from normal temperature, and consecutive days of high heat.

**Method:**

We analyzed daily firearm violence incidents in Syracuse, New York from 2010–2022 using Generalized Linear Autoregressive Moving Average models. Daily maximum temperature, and daily deviation from normal temperature were directly obtained from the National Centers for Environmental Information. Daily apparent maximum temperature was created by combining daily maximum temperature with humidity data derived from windchill and heat index calculations. Consecutive days of high heat was calculated using daily maximum temperature and counting the number of successive days above 90 degrees Fahrenheit (32.2 degrees Celsius). We examined both univariate models for each temperature metric and multivariate models containing multiple temperature variables simultaneously.

**Results:**

Over the 12-year period, Syracuse averaged nearly two firearm violence incidents per day. In univariate analyses, all temperature metrics demonstrated statistically significant relationships with daily firearm violence, with incidence rate ratios between 1.01 and 1.02 per degree Fahrenheit increase (1.02 and 1.04 per degree Celsius increase). However, multivariate analyses revealed more complex patterns. When daily maximum temperature was included with other metrics, it remained the only significant predictor, with each standard deviation increase associated with a 25% increase in daily incidents. When daily apparent maximum temperature replaced daily maximum temperature, daily deviation from normal temperature became the sole significant predictor, with each standard deviation increase associated with a 12% increase in incidents.

**Conclusions:**

Findings suggest different temperature dimensions might be related to firearm violence through distinct pathways. Absolute temperature effects and relative temperature deviations represent separate constructs that independently contribute to weather-gun violence relationships. This complexity supports the need for comprehensive monitoring systems that track both extreme temperatures and departures from seasonal norms to identify periods of elevated risk.

## Introduction

Firearm violence is a significant public health problem in the United States. Nearly half a million deaths between 2010–2020 were the result of gun violence [1]. During this same time period, over one million people were seen in the emergency department for non-fatal, firearm-related injuries [2]. Taken together, these statistics highlight the extensive scope of firearm violence: during a single decade, the number of Americans killed or injured by a firearm exceeded the entire population of Philadelphia, PA, the sixth largest city in the United States [3].

Research has identified several physical features of the neighborhood environment that are associated with firearm violence. These include the presence of vacant and abandoned properties, alcohol outlets, tobacco retailers, and restaurants [4–6]. Collectively, these place-based factors offer an indication of where in the physical environment firearm violence is likely to occur. However, it is also important to consider temporal factors that offer insight into when it is likely to happen. According to temperature-aggression theory, ambient temperature represents one such factor because as temperature increases, people tend to engage in more violent, aggressive behavior that could include gun violence [7–9].

Indeed, a growing body of evidence indicates that higher temperatures coincide with more firearm violence. Kieltyka et al. [10] and Michel et al. [11] both documented links between daily maximum temperature and increased firearm violence in Chicago and Baltimore, respectively. Reeping and Hemenway [12] built upon this work by examining both daily maximum temperature and deviation from normal temperature in Chicago. Their findings suggested that higher temperatures and temperatures deviating from normal historical averages were both associated with increased incidents of gun violence. By demonstrating a link with deviation from normal temperature, these authors showed that it is not only the absolute temperature on a given day that is related to firearm violence but also the degree to which the absolute temperature on a given day represents a stark contrast with what usually occurs.

Broadening the geographical scope of this research, Lyons et al. [13] examined 100 U.S. cities and found that elevated daily maximum temperature is consistently associated with higher rates of firearm violence across different urban settings, suggesting the temperature-firearm violence relationship generalizes across different city settings. More recently, Bushover et al. [14] considered daily maximum temperature, deviation from normal temperature, and the crossed effects of these temperature characteristics. Their findings revealed that days characterized by higher maximum temperature and greater positive deviations from normal temperatures were characterized by elevated rates of firearm violence.

Most recently, Heo et al. [15] analyzed 44 cities across 33 U.S. states. Their study identified associations between daily mean temperature and increased firearm violence, revealing a non-linear relationship where risk levels off at extremely high temperatures. Additionally, they found that cities experiencing greater temperature anomalies (deviations from normal temperature) showed higher rates of gun violence, implicating climate change as an exacerbating factor through increased temperature variability.

While these studies provide evidence for a consistent relationship between temperature and firearm violence, they are limited by their narrow focus on only one or two temperature metrics, typically daily maximum or daily deviation from normal temperature. However, there are other temperature formulations that might better model the relationship between temperature and firearm violence or offer complementary insights into this relationship.

Apparent temperature is a general construct representing the perceived temperature experienced by the human body, derived by adjusting air temperature for the effects of humidity and wind [16,17]. While apparent temperature is often calculated from mean daily temperature readings, it can be equivalently applied to maximum or minimum temperature values using the same underlying equations [18]. Thus, daily apparent maximum temperature refers to the highest felt temperature on a given day. It might better capture the relationship between high temperatures and firearm violence because it maps more directly to the physiological discomfort that drives aggression according to temperature-aggression theory.

Aside from daily apparent maximum temperature, consecutive days of high heat might also be an important temperature metric to consider. As this number gets higher, it could lead to chronic physiological stress, fatigue, and potentially reduced coping capacities. Alternatively, if it has been exceedingly hot for several days, people might adjust, public health messaging might encourage people to stay indoors, and firearm violence might be reduced, suggesting a non-linear effect [15], as well as a potential interaction between consecutive days of high heat and daily maximum temperature, such that the effect of consecutive days of high heat on firearm violence may depend on the temperature reached on a given day.

The purpose of the current study is to explore these possibilities by examining and comparing the predictive capacities of daily maximum temperature, daily apparent maximum temperature, daily deviation from normal temperature, and consecutive days of high heat on daily firearm violence. A summary of the study’s hypotheses is presented in Table 1. Based upon prior research [10–12], we predict that there will be a positive relationship between firearm violence and daily maximum temperature (**hypothesis 1**) as well as daily deviation from normal temperature (**hypothesis 2**). Furthermore, we predict that there will be a positive relationship between daily apparent maximum temperature and firearm violence (**hypothesis 3**) and that daily apparent maximum temperature will have the strongest relationship with gun violence relative to the other temperature metrics, given the closer correspondence between daily apparent maximum temperature and the physiological mechanisms responsible for the temperature-firearm violence relationship according to temperature-aggression theory [7,16,17] (**hypothesis 4**). In addition, we predict that there will be a positive association between consecutive days of high heat and firearm violence (**hypothesis 5**) and that this positive association will be qualified by a non-linear relationship such that the positive association will level off at higher values of consecutive days of high heat [15] (**hypothesis 6**). Finally, we predict that there will be an interaction between consecutive days of high heat and daily maximum temperature such that as the number of consecutive days of high heat increases, the relationship between daily maximum temperature and firearm violence will decrease (**hypothesis 7**).

**Table 1:** Summary of the hypotheses tested.

Hypothesis	Description
1	There will be a positive relationship between firearm violence and daily maximum temperature
2	There will be a positive relationship between firearm violence and daily deviation from normal temperature
3	There will be a positive relationship between firearm violence and daily apparent maximum temperature
4	Daily apparent maximum temperature will have the strongest relationship with gun violence compared to the other temperature metrics
5	There will be a positive association between firearm violence and consecutive days of high heat.
6	There will be a non-linear relationship between firearm violence consecutive days of high heat: the positive association will level off at higher values of consecutive days of high heat
7	There will be an interaction between consecutive days of high heat and daily maximum temperature: as the number of consecutive days of high heat increases, the relationship between daily maximum temperature and firearm violence will decrease

## Method

### Ethics

Syracuse University’s Office of Research Integrity and Protections evaluated the study’s protocol and determined that it did not require institutional review board oversight because all data were de-identified and publicly available.

### Study design

We report this study in accordance with the Strengthening the Reporting of Observational Studies in Epidemiology (STROBE) guidelines [19]. A completed STROBE checklist, with corresponding page and line numbers, is provided in the S1 Checklist in S1 File. We employed an ecological study design that used observational, archival data collected over a 12-year period to examine the relationship between four temperature metrics and daily firearm violence.

### Study setting

Syracuse, NY is a mid-size city located in Central New York. According to the 2020 US Decennial Census, it has a population of 148,620 people, with approximately 46% identifying as White. The median household income in Syracuse is $47,525 and approximately 32% live in poverty. Firearm violence is a long-standing problem in Syracuse’s neighborhoods that has been worsening in recent years [20–22].

### Data

Both the firearm violence data and the weather data reported in this study are available at the Institute for Firearm Injury Prevention Data Repository maintained by the Open Inter-university Consortium for Political and Social Research (https://doi.org/10.3886/E237846V1) [23].

### Outcome

Firearm violence data occurring from 2010–2022 was obtained from the Central New York Crime Analysis Center. We received and first accessed the outcome data on 01-20-2023. The data were provided in a spreadsheet that listed whether an episode of firearm violence had occurred each day between 2010–2020. A firearm episode was defined as an instance in which a gun shot was reported, regardless of whether the gun shot resulted in one or more injuries. The data did not include any identifying information about the people involved in a firearm violence incident. For each day occurring within this date range, we calculated the total number of gun violence incidents that occurred, yielding a daily measure of firearm violence.

### Exposures

**Daily Maximum Temperature** in degrees Fahrenheit was obtained for each day from an online database maintained by the National Centers for Environmental Information (NCEI) [24]. This temperature metric – and the temperature metrics derived from it reported below – were downloaded from the NCEI on 07-12-2024.

**Daily Apparent Maximum Temperature** in degrees Fahrenheit was calculated for each day using maximum temperature data derived from the NCEI [24]. For days when the maximum temperature was < 50 degrees Fahrenheit (< 10.0 degrees Celsius), we used the windchill formula described by the National Weather Service to adjust each maximum temperature value occurring during the study’s focal period [25]. For days when the maximum temperature was > 80 degrees Fahrenheit (> 26.7 degrees Celsius), we used the Rothfusz heat index equation, applying the adjustment to each maximum temperature value when relative humidity exceeded 85% [26].

**Daily Deviation from Normal Temperature** in degrees Fahrenheit was obtained for each day using an online database maintained by the National Centers for Environmental Information (NCEI) [24]. For each calendar date, NCEI calculates a “normal” temperature based on the historical average daily temperature for that same date over the 30-year reference period from 1981–2010. The deviation value represents the difference, in degrees, between the observed daily average temperature and this historical normal, with positive values indicating warmer-than-normal days and negative values indicating cooler-than-normal days.

**Consecutive Days of High Heat** was calculated by creating a running total variable that reflected the number of consecutive days in which the maximum temperature was > 90 degrees Fahrenheit (> 32.2 degrees Celsius). We selected this threshold because the National Weather Service marks it as the temperature required for a day to be counted as part of a “heatwave” in Central New York [27].

### Data analysis

To assess the temporal association between temperature-related variables and daily firearm violence, we used Generalized Linear Autoregressive Moving Average (GLARMA) models via the *glarma* package [28] in R version 4.3.3 [29].. GLARMA models are suitable for count data exhibiting serial correlation, and they enable time-series regression with structured autoregressive terms, offering a flexible extension of generalized linear models for temporally dependent data. Our primary outcome variable was the number of firearm violence incidents per day. All models tested were fit using a negative binomial distribution to accommodate overdispersion in the outcome data, and parameter estimation was performed via Newton-Raphson optimization with Pearson residuals to evaluate model fit. The analysis was structured around a series of GLARMA models to estimate the effects of the different temperature metrics: daily maximum temperature, daily apparent maximum temperature, daily deviation from normal temperature, and consecutive days of high heat. We performed unadjusted univariate and multivariate analyses, as well as covariate adjusted analyses that included annual estimates of median age, unemployment rate, and percentage of males in Syracuse, obtained from the U.S. Census Bureau’s American Community Survey via the *tidycensus* package [30]. Prior to estimating the multivariate models, we screened zero-order correlations among the temperature metrics for potential multicollinearity. Because no single correlation threshold definitively establishes multicollinearity, we treated values of approximately 0.70 or higher as a signal warranting further scrutiny rather than as conclusive evidence of a problem [31]. For any pair of variables that exceeded this screen, we assessed whether the correlation posed an actual estimation problem by fitting a single model containing both metrics and comparing its coefficients and standard errors to those from models that included each metric separately. We treated substantial inflation of the standard errors, or instability in the coefficients such as a change in sign, as indicating collinearity severe enough to preclude joint estimation, in which case we modeled the affected metrics in separate multivariate models.

To test for a non-linear relationship between consecutive days of high heat and daily firearm violence, we estimated models that included both a linear and a quadratic term for consecutive days of high heat. This specification allowed us to assess whether the effect of prolonged heat exposure on firearm violence diminishes or reverses at higher values. To test whether the effect of consecutive days of high heat on firearm violence depends on the hotness of those consecutive days, we estimated models that included an interaction term between consecutive days of high heat and daily maximum temperature, along with both constituent main effects. These models were estimated in both unadjusted and adjusted specifications following the procedures described above.

To control for non-linear trends in firearm violence owing to acute fluctuation occurring around the time of the COVID-19 pandemic [32,33], we used a piecewise discontinuous model of change approach in all models tested (i.e., spline approach). Specifically, we included two linear time trend covariates — one representing pre-pandemic years (2010–2019) and one representing post-pandemic years (2020–2022) — that allowed the slope of the trend in firearm violence to differ across these periods while estimating temperature effects across the full 12-year time series. We addressed seasonality effects using sinusoidal terms (sine and cosine transformations of day-of-year) to model annual cyclical patterns in violence incidence [20].

In all models tested, we implemented the GLARMA models in two stages to address temporal dependence in the time-series data. Initial models were fit without serial dependence terms, serving as a baseline for exploring the relationship between daily firearm violence and the temperature-related variables. Diagnostic autocorrelation function (ACF) and partial autocorrelation function (PACF) plots of the baseline residuals were used to assess serial dependence. Autocorrelations falling outside the white-noise confidence bounds of ±1.96/√n were taken as evidence of residual dependence [34]. We found that each model tested exceeded this criterion on at least 9 lags (range: 9–13), indicating that the assumption of independent errors was violated in all the initial models. To account for this autocorrelation, we incorporated moving average (MA) terms at selected lags, which regress the current noise process on past Pearson residuals. Although the GLARMA framework accommodates both autoregressive (AR) and moving average (MA) components, we limited our specifications to MA terms only. This is standard practice in applied GLARMA work because pure MA specifications are simpler to fit and avoid the identifiability constraints that arise when AR and MA lags are estimated jointly [28]. MA lag structures were selected independently for each model based on inspection of ACF and PACF plots of model residuals, with the goal of eliminating residual autocorrelation. The resulting ACF and PACF plots indicated that the selected MA terms fully resolved the residual autocorrelation present in the time series, confirming the adequacy of the model structure for the time-series nature of the data (see S1-S5 Figs S2 File). To facilitate interpretation, we exponentiated regression coefficients into incidence rate ratios (IRRs) in all models, and for statistically significant temperature metrics in the multivariate models, we calculated the percentage change in daily firearm violence associated with a one standard deviation increase as exp(*b* × *SD*) − 1, holding all other terms constant [35].

## Results and discussion

### Preliminary analyses

Overall, 74/4748 (1.6%) days were missing one or more of the daily temperature variables. Of these, 73 were missing data on only one variable, relative humidity, and 1 was missing data on both daily maximum temperature and daily deviation from normal temperature. However, relative humidity entered our analyses solely as an input to daily apparent maximum temperature, and only through the heat index applied on days exceeding 80 degrees Fahrenheit (26.7 degrees Celsius). All 73 humidity-missing days occurred on cooler days (maximum temperature ≤ 77 degrees Fahrenheit/25.0 degrees Celsius) and thus humidity was never needed to compute apparent maximum temperature on any day it was missing. Consequently, the actual amount of missing data was effectively a single day (1/4748 or 0.02%). Given this negligible proportion of missingness, well below conventional thresholds at which missing data are expected to bias statistical inferences [36,37], listwise deletion was appropriate.

### Descriptive statistics and bivariate correlations

Table 2 presents summary statistics for daily firearm violence and each of the temperature metrics, overall and seasonally. Between 2010–2022, there were nearly 2 episodes of firearm violence per day in the City of Syracuse, with seasonal variation present such that more episodes tended to occur during the warmer months of the year.

**Table 2:** Summary Statistics for Daily Firearm Violence and Temperature Metrics: Overall and Seasonal Estimates, Syracuse, NY (2010–2022).

Variable	Mean	Median	Minimum	Maximum	Standard Deviation
Daily Firearm Violence Overall Winter (Dec21 to Mar19) Spring (Mar20 to Jun20) Summer (Jun21 to Sep21) Fall (Sep22 to Dec20)	1.54 1.08 1.54 1.79 1.72	1.00 1.00 1.00 1.00 1.00	0.00 0.00 0.00 0.00 0.00	11.00 11.00 9.11 10.00 10.00	1.64 1.34 1.64 1.74 1.70
Daily Maximum Temperature Overall Winter (Dec21 to Mar19) Spring (Mar20 to Jun20) Summer (Jun21 to Sep21) Fall (Sep22 to Dec20)	59.16 (15.09) 35.74 (2.08) 64.81 (18.23) 81.20 (27.33) 53.73 (12.07)	61.00 (16.11) 35.00 (1.67) 66.00 (18.89) 82.00 (27.78) 54.00 (12.22)	4.00 (−15.56) 4.00 (−15.56) 21.00 (−6.11) 57.00 (13.89) 15.00 (−9.44)	101.00 (38.33) 80.00 (26.67) 97.00 (36.11) 101.00 (38.33) 91.00 (32.78)	20.85 (11.58) 12.54 (6.97) 14.96 (8.31) 7.10 (3.94) 14.76 (8.20)
Daily Apparent Maximum Temperature Overall Winter (Dec21 to Mar19) Spring (Mar20 to Jun20) Summer (Jun21 to Sep21) Fall (Sep22 to Dec20)	56.46 (13.59) 24.36 (−4.24) 63.70 (17.61) 87.01 (30.56) 48.88 (9.38)	60.44 (15.80) 23.32 (−4.82) 65.71 (18.73) 86.01 (30.01) 52.06 (11.14)	−20.42 (−29.12) −20.42 (−29.12) 2.75 (−16.25) 56.30 (13.50) −4.88 (−20.49)	133.78 (56.54) 82.71 (28.17) 128.27 (53.48) 133.78 (56.54) 116.24 (46.80)	28.91 (16.06) 17.43 (9.68) 19.94 (11.08) 12.63 (7.02) 20.21 (11.23)
Daily Deviation from Normal Temperature Overall Winter (Dec21 to Mar19) Spring (Mar20 to Jun20) Summer (Jun21 to Sep21) Fall (Sep22 to Dec20)	1.71 (0.95) 1.46 (0.81) 1.50 (0.83) 2.22 (1.23) 1.65 (0.92)	1.60 (0.89) 1.80 (1.00) 1.00 (0.56) 2.50 (1.39) 1.10 (0.61)	−35.60 (−19.78) −35.60 (−19.78) −23.50 (−13.06) −14.30 (−7.94) −27.70 (−15.39)	31.50 (17.50) 31.50 (17.50) 30.90 (17.17) 18.70 (10.39) 29.50 (16.39)	8.46 (4.70) 11.05 (6.14) 8.30 (4.61) 5.62 (3.12) 8.08 (4.49)
Consecutive Days of High Heat Overall Winter (Dec21 to Mar19) Spring (Mar20 to Jun20) Summer (Jun21 to Sep21) Fall (Sep22 to Dec20)	0.06 0.00 0.03 0.21 0.01	0.00 0.00 0.00 0.00 0.00	0.00 0.00 0.00 0.00 0.00	7.00 0.00 3.00 7.00 3.00	0.40 0.00 0.22 0.73 0.11

***Note***. Temperatures are presented in degrees Fahrenheit (degrees Celsius).

Table 3 reports zero-order Pearson correlations among the variables. All correlations were statistically significant. Daily maximum temperature and daily apparent maximum temperature shared the largest correlations with daily firearm violence, followed by daily deviation from normal temperature, and consecutive days of high heat. All temperature metrics had small correlations with daily firearm violence. Notably, daily maximum temperature and daily apparent maximum temperature were highly intercorrelated (*r* = 0.99).

**Table 3:** Zero-Order Correlations Between Daily Firearm Violence and the Temperature Metrics ^a^.

Variable	1	2	3	4	5
1. Daily Firearm Violence	1.00				
2. Daily Maximum Temperature	0.20	1.00			
3. Daily Apparent Maximum Temperature	0.20	0.99	1.00		
4. Daily Deviation from Normal Temperature	0.15	0.49	0.49	1.00	
5. Consecutive Days of High Heat	0.07	0.26	0.30	0.19	1.00

^a^All correlations are statistically significant at *p* < .001.

### GLARMA models and interpretation

Fig 1 presents scatterplots with superimposed loess curves representing the relationship between each temperature metric and daily firearm violence. A relatively linear relationship was present for each predictor, except for consecutive days of high heat, which seemed to plateau at higher values. This observation was consistent with hypothesis 6 predicting that consecutive days of high heat might have a non-linear relationship with firearm violence.

**Fig 1:**
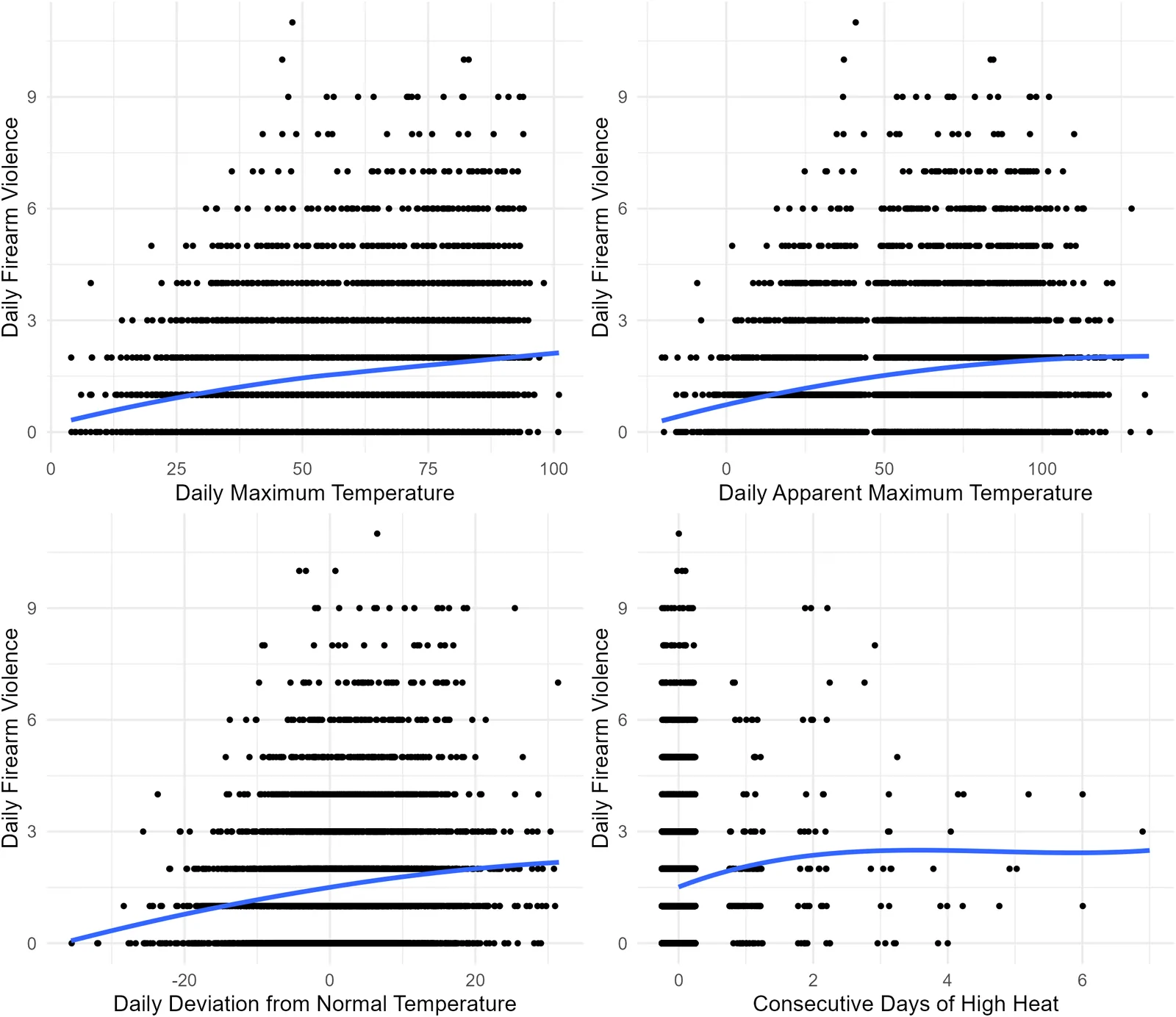
Scatterplots with Loess Curves Displaying the Relationships Between the Temperature Predictors and Daily Firearm Violence. The blue line in each scatterplot is a loess curve representing the relationship between each temperature metric (in degrees Fahrenheit for daily maximum temperature, daily apparent maximum temperature, and daily deviation from normal temperature) and daily firearm violence.

Table 4, Table 5, Table 6, Table 7, Table 8 report the coefficients of the temperature metrics in each of the models tested. Full models containing all covariates (if applicable) and MA terms are available in the Supporting Information (see S1-8 Tables in S2 File).

**Table 4:** Univariate glarma models examining the relationship between daily firearm violence and each temperature metric.

Variable ^a^	Unadjusted				Adjusted ^b^			
	b	IRR	95%CI	p	b	IRR	95%CI	p
Daily Maximum Temperature	0.016	1.016	1.013, 1.019	<.001	0.016	1.016	1.013, 1.019	<.001
Daily Apparent Maximum Temperature	0.010	1.010	1.008, 1.012	<.001	0.010	1.010	1.008, 1.012	<.001
Daily Deviation from Normal Temperature	0.018	1.018	1.015, 1.022	<.001	0.018	1.019	1.015, 1.022	<.001
Consecutive Days of High Heat	0.066	1.068	1.001, 1.139	.045	0.065	1.067	1.000, 1.138	.049

***Note.*** *b* = unstandardized coefficient, *IRR* = incidence rate ratio. *95% CI =* 95 percent confidence interval. Regression coefficients are reported to 3 decimal places to facilitate interpretation.

^a^Coefficients for temperature metrics measured in degrees (daily maximum, daily apparent maximum, and daily deviation from normal) are expressed per-1°Fahrenheit. Corresponding per-1°Celsius incidence rate ratios are obtained by raising each IRR to the power of 1.8; confidence interval limits transform identically and p-values are unchanged. Consecutive days of high heat is measured in days and is not rescaled. Both unadjusted and adjusted models include linear terms representing pre-pandemic years (2010–2019) and post-pandemic years (2020–2022) as well as a sine and cosine transformations of day-of-year to model annual cyclical patterns in violence incidence. Moving average lags differed by temperature metric. Daily maximum temperature: unadjusted = 1–8, 13, 14; adjusted = 1–3, 5–8, 13, 14. Daily apparent maximum temperature: unadjusted = 1–9, 11–14; adjusted = 1–3, 5–8, 13, 14. Daily deviation from normal temperature: unadjusted = 1–8, 13, 14; adjusted = 1–3, 5–8, 13, 14. Consecutive days of high heat: unadjusted = 1–9, 13, 14; adjusted = 1–8, 13, 14.

^b^Adjusted models include annual median age, percentage of males, and unemployment rate.

**Table 5:** Multivariate GLARMA models examining the relationships between daily firearm violence and the temperature metrics.

Variable ^a^	Unadjusted				Adjusted ^b^			
	b	IRR	95% CI	p	b	IRR	95% CI	p
**Model 1**								
Daily Maximum Temperature	0.011	1.011	1.003, 1.018	.004	0.010	1.010	1.003, 1.018	.006
Daily Deviation from Normal Temperature	0.007	1.007	0.998, 1.016	.13	0.007	1.007	0.999, 1.016	.10
Consecutive Days of High Heat	−0.001	0.999	0.936, 1.066	.97	−0.003	0.997	0.935, 1.064	.93
**Model 2**								
Daily Apparent Maximum Temperature	0.003	1.003	0.998, 1.008	.18	0.003	1.003	0.998, 1.008	.24
Daily Deviation from Normal Temperature	0.014	1.014	1.006, 1.022	<.001	0.014	1.014	1.006, 1.023	<.001
Consecutive Days of High Heat	−0.010	0.991	0.927, 1.059	.78	−0.010	0.990	0.927, 1.059	.78

***Note.*** *b* = unstandardized coefficient, *IRR* = incidence rate ratio. *95% CI =* 95 percent confidence interval. Regression coefficients are reported to 3 decimal places to facilitate interpretation.

^a^Coefficients for temperature metrics measured in degrees (daily maximum, daily apparent maximum, and daily deviation from normal) are expressed per-1°Fahrenheit. Corresponding per −1°Celsius incidence rate ratios are obtained by raising each IRR to the power of 1.8; confidence interval limits transform identically and p-values are unchanged. Consecutive days of high heat is measured in days and is not rescaled. Both unadjusted and adjusted models include linear terms representing pre-pandemic years (2010–2019) and post-pandemic years (2020–2022) as well as a sine and cosine transformations of day-of-year to model annual cyclical patterns in violence incidence. Moving average terms were estimated at lags 1, 2, 3, 5, 6, 7, 8, 13, and 14 in all models except unadjusted model 1, which also included a moving average term at lag 4.

^b^Adjusted models include annual median age, percentage of males, and unemployment rate.

**Table 6:** Univariate GLARMA models examining whether a non-linear relationship exists between daily firearm violence and consecutive days of high heat.

Variable ^a^	Unadjusted				Adjusted ^b^			
	b	IRR	95% CI	p	b	IRR	95% CI	p
Consecutive Days of High Heat	0.151	1.163	1.012, 1.337	.034	0.149	1.160	1.010, 1.333	.036
Consecutive Days of High Heat ^ 2	−0.023	0.977	0.944, 1.011	.19	−0.023	0.977	0.945, 1.011	.19

***Note.*** *b* = unstandardized coefficient, *IRR* = incidence rate ratio. *95% CI =* 95 percent confidence interval. Regression coefficients are reported to 3 decimal places to facilitate interpretation.

^a^Both unadjusted and adjusted models include linear terms representing pre-pandemic years (2010–2019) and post-pandemic years (2020–2022) as well as a sine and cosine transformations of day-of-year to model annual cyclical patterns in violence incidence. Moving average terms were estimated at lags 1, 2, 3, 4, 5, 6, 7, 8, 13, and 14 in both the unadjusted and adjusted models.

^b^Adjusted models include annual median age, percentage of males, and unemployment rate.

**Table 7:** Multivariate GLARMA models examining whether a non-linear relationship exists between daily firearm violence and consecutive days of high heat.

Variable ^a^	Unadjusted				Adjusted ^b^			
	b	IRR	95% CI	p	b	IRR	95% CI	p
Daily Maximum Temperature	0.011	1.011	1.004, 1.018	.004	0.010	1.010	1.003, 1.018	.006
Daily Deviation from Normal Temperature	0.007	1.007	0.998, 1.016	.125	0.007	1.007	0.999, 1.016	.100
Consecutive Days of High Heat	0.003	1.003	0.873, 1.152	.968	0.003	1.003	0.873, 1.152	.969
Consecutive Days of High Heat ^ 2	−0.001	0.999	0.966, 1.033	.942	−0.002	0.998	0.966, 1.032	.928

***Note.*** *b* = unstandardized coefficient, *IRR* = incidence rate ratio. *95% CI =* 95 percent confidence interval. Regression coefficients are reported to 3 decimal places to facilitate interpretation.

^a^Coefficients for temperature metrics measured in degrees (daily maximum, daily apparent maximum, and daily deviation from normal) are expressed per-1°Fahrenheit. Corresponding per −1°Celsius incidence rate ratios are obtained by raising each IRR to the power of 1.8; confidence interval limits transform identically and p-values are unchanged. Consecutive days of high heat is measured in days and is not rescaled. Both unadjusted and adjusted models include linear terms representing pre-pandemic years (2010–2019) and post-pandemic years (2020–2022) as well as a sine and cosine transformations of day-of-year to model annual cyclical patterns in violence incidence. Moving average terms were estimated at lags 1, 2, 3, 5, 6, 7, 8, 13, and 14 in both the unadjusted and adjusted models.

^b^Adjusted models include annual median age, percentage of males, and unemployment rate.

**Table 8:** Multivariate GLARMA model examining whether an interaction exists between daily maximum temperature and consecutive days of high heat on daily firearm violence.

Variable ^a^	Unadjusted				Adjusted ^b^			
	b	IRR	95% CI	p	b	IRR	95% CI	p
Daily Maximum Temperature	0.011	1.011	1.004, 1.018	.004	0.010	1.010	1.003, 1.018	.006
Daily Deviation from Normal Temperature	0.007	1.007	0.998, 1.016	0.13	0.007	1.007	0.999, 1.016	.10
Consecutive Days of High Heat	1.229	3.418	0.235, 49.686	0.37	1.150	3.158	0.220, 45.310	.40
Consecutive Days of High Heat ^ 2	0.008	1.008	0.969, 1.048	0.69	0.007	1.007	0.969, 1.047	.72
Daily Maximum Temperature x Consecutive Days of High Heat	−0.014	0.987	0.958, 1.016	0.37	−0.013	0.987	0.959, 1.017	.40

***Note.*** *b* = unstandardized coefficient, *IRR* = incidence rate ratio. *95% CI =* 95 percent confidence interval. Regression coefficients are reported to 3 decimal places to facilitate interpretation.

^a^Coefficients for temperature metrics measured in degrees (daily maximum, daily apparent maximum, and daily deviation from normal) are expressed per-1°Fahrenheit. Corresponding per-1°Celsius incidence rate ratios are obtained by raising each IRR to the power of 1.8; confidence interval limits transform identically and p-values are unchanged. Consecutive days of high heat is measured in days and is not rescaled. The daily maximum temperature × consecutive days of high heat interaction converts in the same way. Both unadjusted and adjusted models include linear terms representing pre-pandemic years (2010–2019) and post-pandemic years (2020–2022) as well as a sine and cosine transformations of day-of-year to model annual cyclical patterns in violence incidence. Moving average terms were estimated at lags 1, 2, 3, 5, 6, 7, 8, 13, and 14 in both the unadjusted and adjusted models.

^b^Adjusted models include annual median age, percentage of males, and unemployment rate.

In both the unadjusted and covariate adjusted univariate GLARMA models, we replicated prior research showing a statistically significant association between daily maximum temperature and firearm violence [10,11,13] – see Table 4. This is consistent with hypothesis 1. The IRRs in both the unadjusted and covariate adjusted analyses indicates that each 1-degree Fahrenheit increase in maximum temperature is associated with a roughly 2% higher likelihood of firearm violence on that day (equivalently, each 1-degree Celsius increase is associated with an approximately 3% increase). This finding is consistent with temperature-aggression theory predictions [7]. Statistically significant positive associations between the other temperature metrics and daily firearm violence were also observed, consistent with hypotheses 2, 3, and 5.

Prior to interpreting the multivariate models, we examined whether the high correlation between daily maximum temperature and daily apparent maximum temperature (*r* = 0.99) posed an estimation problem by fitting a single model containing both metrics and comparing it to models that included each metric separately. In the combined model, the standard errors of both temperature coefficients were approximately double those in the separate models, and the apparent temperature coefficient reversed direction, shifting from a non-significant positive association in its separate model to a significant negative one when estimated alongside daily maximum temperature. This pattern is characteristic of unstable estimation under collinearity [31]. We therefore modeled the two metrics separately.

Table 5 reports the results of the multivariate models, which revealed more nuanced patterns when the temperature metrics were considered simultaneously. In model 1, daily maximum temperature was the only temperature metric significantly related to daily firearm violence in both unadjusted and covariate adjusted analyses. Each 1 SD increase in daily maximum temperature was associated with an approximately 25% increase in the number of daily firearm violence episodes. Neither daily deviation from normal temperature nor consecutive days of high heat were statistically significantly related to daily firearm violence. A different pattern emerged when daily apparent maximum temperature replaced daily maximum temperature in model 2. In this model, daily deviation from normal temperature was the only statistically significant temperature predictor in both the unadjusted and covariate adjusted analyses. Each 1 SD increase in daily deviation was associated with approximately a 12% increase in the number of daily shootings. Neither daily apparent maximum temperature nor consecutive days of high heat were related to daily shootings. Collectively, these results are counter to the prediction made by hypothesis 4.

These findings initially appear surprising because one might expect daily maximum and daily apparent maximum temperature to function similarly in each model given their high collinearity. Indeed, a majority of their variance is shared. However, this expectation overlooks the 1% of non-shared variance between them, which appears to be meaningful for understanding their differential relationships with daily firearm violence. Daily apparent maximum temperature incorporates information about humidity, and humidity tends to exacerbate during heatwaves, i.e., consecutive days of high heat; [38,39]. This tendency might be leading to the cancellation of these temperature metrics when they are included in the same model.

The differential significance of daily maximum temperature and daily deviation from normal temperature in separate models suggests that distinct mechanisms might underpin the relationship each shares with daily firearm violence. Daily maximum temperature likely taps into the discomfort that accompanies rising absolute temperatures, creating physiological and perceptional changes that might trigger aggressive responses [9,40,41]. Conversely, daily departure from normal temperature likely captures the jarring impact that sudden temperature deviations can have on behavior and social routines given sudden temperature deviations. Indeed, prior evidence suggests that temperature deviations are relevant to firearm violence [14,15]. The significance of this metric when included with apparent maximum temperature and consecutive days of high heat indicates that the unusualness of a particularly hot day may be more important for predicting firearm violence than the humidity that accompanies elevated temperature or the cumulative effect of preceding hot days. This finding suggests that relative, rather than absolute, temperature changes may be particularly disruptive to normal behavioral patterns and social functioning.

Overall, these results indicate that there is no single best predictor of daily firearm violence among the temperature metrics evaluated. Instead, our findings suggest that these different metrics likely capture different aspects of temperature that relate to firearm violence through potentially distinct pathways. This complexity highlights the multifaceted nature of temperature effects on human behavior and suggests that comprehensive models of weather-violence relationships should consider multiple dimensions of meteorological variation rather than relying on single temperature measures.

In the univariate model examining whether a non-linear relationship existed between consecutive days of high heat and daily firearm violence, the quadratic term was not statistically significant in either the unadjusted (IRR = 0.977, *p* = .19) or covariate adjusted (IRR = 0.977, *p* = .19) analyses (see Table 6). Although the negative direction of the quadratic coefficient is consistent with a plateau or diminishing effect at higher values of consecutive heat days, as suggested by the loess curve in Fig 1, the evidence for a non-linear relationship was not statistically significant. When this non-linear term was incorporated into the multivariate model alongside daily maximum temperature and daily deviation from normal temperature, neither the linear nor the quadratic term for consecutive days of high heat was statistically significant in either the unadjusted or covariate adjusted analyses (see Table 7). Collectively, these results are contrary to hypothesis 6. Daily maximum temperature remained the only significant predictor (unadjusted IRR = 1.011, *p* = .004; adjusted IRR = 1.010, *p* = .006), consistent with the pattern observed in the linear multivariate models reported in Table 5.

Finally, to test whether the effect of consecutive days of high heat depends upon the temperature of those consecutive days, we added an interaction term between consecutive days of high heat and daily maximum temperature to the non-linear multivariate model. The interaction was not statistically significant in either the unadjusted (IRR = 0.987, *p* = .369) or covariate adjusted (IRR = 0.987, *p* = .398) analyses (see Table 8). This is contrary to hypothesis 7. Taken together, these analyses suggest that consecutive days of high heat does not exhibit a reliably non-linear relationship with daily firearm violence, nor does it moderate the effect of daily maximum temperature, at least within the constraints of this sample.

Even though the quadratic term for consecutive days of high heat was not statistically significant, the negative direction of its coefficient and the visual pattern in Fig 1 are consistent with a broader body of literature documenting curvilinear temperature-violence relationships. Heo et al. [15] found that the risk of violent crime increased with temperature up to approximately the 90th percentile but decreased thereafter, producing an inverted J-shaped exposure-response curve across 44 U.S. cities. Similarly, Berman et al. [42] observed that crime risk plateaued and declined at high daily temperatures across 436 U.S. counties, and Stevens et al. [43] reported that the rate of increase in assault counts slowed as temperatures exceeded 30°C in New South Wales, Australia. Choi et al. [8] noted in their systematic review that several studies have documented decreases in crime risk during extreme heat relative to moderate-to-high temperature levels. The visual plateau observed in Fig 1 for consecutive days of high heat may reflect a similar phenomenon applied to cumulative heat exposure rather than single-day temperature extremes.

Notably, this pattern may not have reached statistical significance in our data because days with consecutive high heat were rare in our sample; consecutive days of high heat had a mean of only 0.06, indicating that the vast majority of study days had a value of zero on this variable. This heavy zero-inflation substantially limits the effective sample size available to detect non-linear effects at higher values. Similarly, it might also have limited our ability to detect an interaction between consecutive days of high heat and daily maximum temperature. It is possible that the non-linear and interactive patterns would emerge more clearly in locations with longer and more frequent heatwaves or in multi-city studies with greater variation in sustained heat exposure. Future research should examine whether non-linear and interactive effects of cumulative heat on firearm violence are detectable in warmer climatic contexts with more frequent extended heat events.

### Limitations

Our findings should be interpreted in light of several limitations. First, although Syracuse represents an important study setting given the elevated firearm violence that occurs there, it is a single geographic location, constraining our ability to generalize our findings. Future research should replicate our approach using cities at varied locations. Second, our study timeframe was limited to 12 years and thus cannot yield insight into the effects that longer-term temperature trends might have on firearm violence. For example, contemporary climate change is leading to more erratic weather patterns and might be contributing to the poignant effects of daily deviation from normal temperature relative to the other temperature metrics included in our models. Third, our observational study design limits our ability to draw causal inferences from our findings. Future research should utilize laboratory-based models that experimentally manipulate the different aspects of temperature we examined to determine their effects on human behavior in a controlled environment. Finally, our models do not account for spatial heterogeneity. It is possible that the relationship between temperature and firearm violence varies according to place-based features [4–6]. Future research should examine whether the temperature metrics we examined might relate to firearm violence in different ways depending upon the presence of these place-based features.

## Conclusion

In sum, our research advances our understanding of the temperature-firearm violence relationship by demonstrating that different dimensions of heat exposure influence daily firearm violence likely through different pathways. Our findings suggest that absolute temperature effects (daily maximum temperature) and relative temperature effects (daily deviation from normal temperature) represent separate constructs that independently contribute to understanding weather-violence relationships. The complexity revealed in our multivariate analysis supports a more nuanced theoretical framework that acknowledges multiple heat-related stressors. These findings have important implications for public health surveillance and violence prevention efforts, as they suggest that monitoring systems should track both extreme temperatures and departures from seasonal norms to identify periods of elevated risk. Understanding these distinct temperature pathways may inform more targeted interventions during meteorologically high-risk periods.

## Supporting information

S1 File STROBE Checklist. (DOCX)

S2 File Supplementary Figures and Tables. (DOCX)
