# Supplementary material for: Examining the relationship between temperature and firearm violence: A comparison of metrics
Source: PLoS One. 2026 Sep 30;21(9):e0358196. doi: 10.1371/journal.pone.0358196 (PMC13626328; doi:10.1371/journal.pone.0358196)
Supplement: Supplementary file 1 [file pone.0358196.s001.docx]

**Checklist S1. Strengthening the Reporting of Observational Studies in Epidemiology (STROBE) Checklist**

|  | Item No. | Recommendation | Page  No. | Relevant text from manuscript |
| --- | --- | --- | --- | --- |
| **Title and abstract** | 1 | (*a*) Indicate the study’s design with a commonly used term in the title or the abstract | p. 1–2 | We analysed daily firearm violence incidents in Syracuse (2010–2022) using GLARMA models. |
|  |  | (*b*) Provide in the abstract an informative and balanced summary of what was done and what was found | p. 2-3 | Abstract summarizes objectives, design, statistical methods (GLARMA), main findings (significant temperature–violence relationships), and conclusions. |
| Introduction | | | |  |
| Background/rationale | 2 | Explain the scientific background and rationale for the investigation being reported | p. 4-7 | Introduction describes firearm violence as a major public-health problem and explains temperature-aggression theory as scientific rationale for the relationship between ambient temperature and firearm violence. |
| Objectives | 3 | State specific objectives, including any prespecified hypotheses | p. 6-7 | Objective: to compare four temperature metrics (max, apparent max, deviation, consecutive heat days) as predictors of firearm violence. |
| Methods | | | |  |
| Study design | 4 | Present key elements of study design early in the paper | p. 6 | We employed an ecological study design that used observational, archival data collected over a 12-year period to examine the relationship between four temperature metrics and daily firearm violence. |
| Setting | 5 | Describe the setting, locations, and relevant dates, including periods of recruitment, exposure, follow-up, and data collection | p. 7-10 | Study conducted in Syracuse, NY, a mid-size city (population 148,620; 32% below poverty). Firearm violence is a persistent local problem.  Firearm violence data occurring from 2010-2022 was obtained from the Central New York Crime Analysis Center |
| Participants | 6 | (*a*) *Cohort study*—Give the eligibility criteria, and the sources and methods of selection of participants. Describe methods of follow-up  *Case-control study*—Give the eligibility criteria, and the sources and methods of case ascertainment and control selection. Give the rationale for the choice of cases and controls  *Cross-sectional study*—Give the eligibility criteria, and the sources and methods of selection of participants | p. 7-8 | All firearm incidents in Syracuse (2010–2022) included; daily totals calculated from Central NY Crime Analysis Center records; no eligibility restrictions. |
|  |  | (*b*) *Cohort study*—For matched studies, give matching criteria and number of exposed and unexposed  *Case-control study*—For matched studies, give matching criteria and the number of controls per case | N/A | N/A |
| Variables | 7 | Clearly define all outcomes, exposures, predictors, potential confounders, and effect modifiers. Give diagnostic criteria, if applicable | p. 8-10 | Outcome: daily firearm incidents. Exposures: four temperature metrics—maximum, apparent maximum, deviation from normal, and consecutive high-heat days (>90 °F threshold). |
| Data sources/ measurement | 8* | For each variable of interest, give sources of data and details of methods of assessment (measurement). Describe comparability of assessment methods if there is more than one group | p. 8-10 | Firearm data from Central New York Crime Analysis Center; temperature variables from National Centers for Environmental Information; apparent temperature adjusted using windchill and heat-index equations; high-heat defined as >90 °F per National Weather Service |
| Bias | 9 | Describe any efforts to address potential sources of bias | N/A | N/A |
| Study size | 10 | Explain how the study size was arrived at | p. 8; 13 | Full daily dataset for 2010–2022 analyzed after removing missing data. |

Continued on next page

| Quantitative variables | 11 | Explain how quantitative variables were handled in the analyses. If applicable, describe which groupings were chosen and why | p. 10-12 | Daily firearm incidents modelled as count data using negative binomial GLARMA models. Temperature variables analysed as continuous predictors; regression coefficients exponentiated to incidence rate ratios (IRRs) per 1-SD increase. No grouping or categorization applied. |
| --- | --- | --- | --- | --- |
| Statistical methods | 12 | (*a*) Describe all statistical methods, including those used to control for confounding | p. 10-12 | GLARMA models (negative binomial) used to model count data with temporal autocorrelation. Confounding controlled by including time covariates (pre/post-pandemic trends) and seasonality terms (sine–cosine transformations). |
|  |  | (*b*) Describe any methods used to examine subgroups and interactions | p. 10-12 | Univariate and multivariate GLARMA models run separately for each temperature metric to assess independent and combined effects (no subgroup interactions tested). |
|  |  | (*c*) Explain how missing data were addressed | p. 13 | Days with missing temperature data (1.6%) were excluded from analysis. |
|  |  | (*d*) *Cohort study*—If applicable, explain how loss to follow-up was addressed  *Case-control study*—If applicable, explain how matching of cases and controls was addressed  *Cross-sectional study*—If applicable, describe analytical methods taking account of sampling strategy | N/A | N/A |
|  |  | (*e*) Describe any sensitivity analyses | p. 10-12 | Model sensitivity to autocorrelation was evaluated via residual diagnostics (ACF/PACF). ARMA terms at lags 1–8 and 12–14 added to remove residual autocorrelation; final models showed adequate fit. |
| Results | | | | |
| Participants | 13* | (a) Report numbers of individuals at each stage of study—eg numbers potentially eligible, examined for eligibility, confirmed eligible, included in the study, completing follow-up, and analysed | p. 8, 13 | Daily firearm violence data from 2010–2022 were obtained from the Central New York Crime Analysis Center (~4,748 days). Seventy-four days (1.6%) were missing at least one temperature variable and were excluded. |
|  |  | (b) Give reasons for non-participation at each stage | N/A | N/A |
|  |  | (c) Consider use of a flow diagram | N/A | N/A |
| Descriptive data | 14* | (a) Give characteristics of study participants (eg demographic, clinical, social) and information on exposures and potential confounders | p. 13-16 | Table 2 reports descriptive statistics for daily firearm violence (mean = 1.54 incidents/day) and temperature metrics. |
|  |  | (b) Indicate number of participants with missing data for each variable of interest | p. 13 | Seventy-four days (1.6%) had missing temperature data – 73 missing humidity and 1 missing both maximum and deviation temperatures. |
|  |  | (c) *Cohort study*—Summarise follow-up time (eg, average and total amount) | N/A | N/A |
| Outcome data | 15* | *Cohort study*—Report numbers of outcome events or summary measures over time | p. 13 | Between 2010–2022, there was approximately 1 episode of firearm violence per day in the City of Syracuse. Table 2 reports the mean (1.54), median (1.00), minimum (0), and maximum (11) of daily firearm incidents. Univariate and multivariate GLARMA models (Tables 3–4) report incidence rate ratios (IRRs) quantifying the association between temperature metrics and daily firearm violence. |
|  |  | *Case-control study—*Report numbers in each exposure category, or summary measures of exposure | N/A | N/A |
|  |  | *Cross-sectional study—*Report numbers of outcome events or summary measures | N/A | N/A |
| Main results | 16 | (*a*) Give unadjusted estimates and, if applicable, confounder-adjusted estimates and their precision (eg, 95% confidence interval). Make clear which confounders were adjusted for and why they were included | p. 17-28 | Univariate GLARMA models showed all temperature metrics significantly associated with firearm violence (IRRs ≈ 1.01–1.02). Multivariate models: 1-SD higher maximum temperature → 16% increase; 1-SD higher deviation from normal → 31% increase. |
|  |  | (*b*) Report category boundaries when continuous variables were categorized | N/A | N/A |
|  |  | (*c*) If relevant, consider translating estimates of relative risk into absolute risk for a meaningful time period | N/A | N/A |

Continued on next page

| Other analyses | 17 | Report other analyses done—eg analyses of subgroups and interactions, and sensitivity analyses | N/A | N/A |
| --- | --- | --- | --- | --- |
| Discussion | | | | |
| Key results | 18 | Summarise key results with reference to study objectives | p. 17-29 | Findings show that absolute (maximum temperature) and relative (deviation from normal) heat measures independently influence daily firearm violence, meeting study objectives |
| Limitations | 19 | Discuss limitations of the study, taking into account sources of potential bias or imprecision. Discuss both direction and magnitude of any potential bias | p. 28-29 | Limitations include single-city data, 12-year timeframe, and observational design, which restrict generalisability and causal inference. |
| Interpretation | 20 | Give a cautious overall interpretation of results considering objectives, limitations, multiplicity of analyses, results from similar studies, and other relevant evidence | p. 29 | Absolute and relative temperature metrics likely act through distinct behavioural mechanisms; comprehensive models should integrate multiple meteorological dimensions |
| Generalisability | 21 | Discuss the generalisability (external validity) of the study results | p. 28-29 | Results may not generalise beyond Syracuse; replication in other urban contexts is recommended. |
| Other information | |  | | |
| Funding | 22 | Give the source of funding and the role of the funders for the present study and, if applicable, for the original study on which the present article is based | p. 1 | Supported by the National Institute of Minority Health and Health Disparities (R21MD019083-01). Funders had no role in study design, analysis, or interpretation. |

*Give information separately for cases and controls in case-control studies and, if applicable, for exposed and unexposed groups in cohort and cross-sectional studies.

**Note:** An Explanation and Elaboration article discusses each checklist item and gives methodological background and published examples of transparent reporting. The STROBE checklist is best used in conjunction with this article (freely available on the Web sites of PLoS Medicine at http://www.plosmedicine.org/, Annals of Internal Medicine at http://www.annals.org/, and Epidemiology at http://www.epidem.com/). Information on the STROBE Initiative is available at [www.strobe-statement.org](http://www.strobe-statement.org).
