# Supplementary material for: Examining the relationship between temperature and firearm violence: A comparison of metrics
Source: PLoS One. 2026 Sep 30;21(9):e0358196. doi: 10.1371/journal.pone.0358196 (PMC13626328; doi:10.1371/journal.pone.0358196)
Supplement: Supplementary file 2 [file pone.0358196.s002.docx]

# Figure S1. ACF and PACF Plots Associated with the Univariate GLARMA Models Examining the Relationship Between Daily Firearm Violence and Each Temperature Metric.

Note. Dashed lines mark the approximate 95% significance bounds (±1.96/√*N*) for the residual autocorrelations under the null hypothesis of no autocorrelation. Spikes beyond these bounds indicate significant temporal dependence. Moving average terms were added at successive lags until residual autocorrelations fell within the bounds.

# Figure S2. ACF and PACF Plots Associated with Multivariate GLARMA Models Examining the Relationships Between Daily Firearm Violence and the Temperature Metrics.

Note. Dashed blue lines mark the approximate 95% significance bounds (±1.96/√*N*) for the residual autocorrelations under the null hypothesis of no autocorrelation. Spikes beyond these bounds indicate significant temporal dependence. Moving average terms were added at successive lags until residual autocorrelations fell within the bounds.

# Figure S3. ACF and PACF Plots Associated with the Univariate GLARMA Models Examining Whether a Non-Linear Relationship Exists Between Daily Firearm Violence and Consecutive Days of High Heat.

Note. Dashed lines mark the approximate 95% significance bounds (±1.96/√*N*) for the residual autocorrelations under the null hypothesis of no autocorrelation. Spikes beyond these bounds indicate significant temporal dependence. Moving average terms were added at successive lags until residual autocorrelations fell within the bounds.

# Figure S4. ACF and PACF Plots Associated with the Multivariate GLARMA Models Examining Whether a Non-Linear Relationship Exists Between Daily Firearm Violence and Consecutive Days of High Heat

Note. Dashed lines mark the approximate 95% significance bounds (±1.96/√*N*) for the residual autocorrelations under the null hypothesis of no autocorrelation. Spikes beyond these bounds indicate significant temporal dependence. Moving average terms were added at successive lags until residual autocorrelations fell within the bounds.

# Figure S5. ACF and PACF Plots Associated with the Multivariate GLARMA Models Examining Whether an Interaction Exists Between Daily Maximum Temperature and Consecutive Days of High Heat on Daily Firearm Violence.

Note. Dashed lines mark the approximate 95% significance bounds (±1.96/√*N*) for the residual autocorrelations under the null hypothesis of no autocorrelation. Spikes beyond these bounds indicate significant temporal dependence. Moving average terms were added at successive lags until residual autocorrelations fell within the bounds.

# Table S1. Full Univariate GLARMA Models Examining the Relationship Between Daily Firearm Violence and Daily Maximum Temperature.

| **Variable ^a^** | **Unadjusted** | | | | **Adjusted ^b^** | | | |
| --- | --- | --- | --- | --- | --- | --- | --- | --- |
|  | b | 95% CI | IRR | p | b | 95% CI | IRR | p |
| Daily Maximum Temperature | 0.016 | 1.013, 1.019 | 1.016 | <.001 | 0.016 | 1.013, 1.019 | 1.016 | <.001 |
| 2010-2019 | 0.108 | 1.098, 1.130 | 1.114 | <.001 | 0.098 | 1.078, 1.128 | 1.103 | <.001 |
| 2020-2022 | 0.033 | 0.959, 1.114 | 1.034 | .383 | -0.047 | 0.875, 1.041 | 0.954 | .292 |
| Sine | -0.077 | 0.868, 0.987 | 0.925 | .018 | -0.076 | 0.871, 0.987 | 0.927 | .017 |
| Cosine | 0.207 | 1.121, 1.348 | 1.229 | <.001 | 0.210 | 1.127, 1.350 | 1.233 | <.001 |
| Median Age | — | — | — | — | -0.022 | 0.904, 1.058 | 0.978 | .579 |
| Percent Male | — | — | — | — | 0.106 | 1.042, 1.186 | 1.112 | .001 |
| Unemployment Rate | — | — | — | — | -0.036 | 0.935, 0.995 | 0.965 | .023 |
| Alpha | 5.157 | 4.210, 6.104 | — | <.001 | 5.200 | 4.240, 6.160 | — | <.001 |
| Lag 1 | 0.101 | 0.076, 0.127 | — | <.001 | 0.098 | 0.073, 0.124 | — | <.001 |
| Lag 2 | 0.033 | 0.007, 0.059 | — | .014 | 0.029 | 0.004, 0.054 | — | .026 |
| Lag 3 | 0.038 | 0.011, 0.064 | — | .005 | 0.032 | 0.006, 0.058 | — | .016 |
| Lag 4 | 0.026 | -0.000, 0.052 | — | .053 | — | — | — | — |
| Lag 5 | 0.028 | 0.002, 0.055 | — | .036 | 0.025 | -0.002, 0.052 | — | .064 |
| Lag 6 | 0.039 | 0.013, 0.065 | — | .003 | 0.036 | 0.010, 0.062 | — | .007 |
| Lag 7 | 0.082 | 0.056, 0.109 | — | <.001 | 0.080 | 0.053, 0.106 | — | <.001 |
| Lag 8 | 0.049 | 0.022, 0.075 | — | <.001 | 0.047 | 0.020, 0.073 | — | <.001 |
| Lag 13 | 0.023 | -0.004, 0.050 | — | .094 | 0.021 | -0.006, 0.048 | — | .125 |
| Lag 14 | 0.072 | 0.048, 0.096 | — | <.001 | 0.069 | 0.044, 0.093 | — | <.001 |
| **Note**. b = unstandardized coefficient, IRR = incidence rate ratio. 95% CI = 95 percent confidence interval. Em-dashes indicate that a covariate was not included in the model, a moving average lag was not estimated, or that an incidence rate ratio is not interpretable (as is the case for the negative binomial dispersion parameter and moving average lag coefficients). Regression coefficients are reported to 3 decimal places to facilitate interpretation. | | | | | | | | |
| ^a^ The coefficient for daily maximum temperature is expressed per-1°Fahrenheit. A corresponding per -1°Celsius incidence rate ratio is obtained by raising the IRR to the power of 1.8; the confidence interval limits transform identically and p-values are unchanged. Both unadjusted and adjusted models include linear terms representing pre-pandemic years (2010-2019) and post-pandemic years (2020-2022) as well as sine and cosine transformations of day-of-year to model annual cyclical patterns in violence incidence. Moving average terms were estimated at lags 1, 2, 3, 4, 5, 6, 7, 8, 13, and 14 in the unadjusted model, and at lags 1, 2, 3, 5, 6, 7, 8, 13, and 14 in the adjusted model. | | | | | | | | |
| ^b^ Adjusted models include annual median age, percentage of males, and unemployment rate. | | | | | | | | |

# Table S2. Full Univariate GLARMA Models Examining the Relationship Between Daily Firearm Violence and Daily Apparent Maximum Temperature.

| **Variable ^a^** | **Unadjusted** | | | | **Adjusted ^b^** | | | |
| --- | --- | --- | --- | --- | --- | --- | --- | --- |
|  | b | 95% CI | IRR | p | b | 95% CI | IRR | p |
| Daily Apparent Maximum Temperature | 0.010 | 1.008, 1.012 | 1.010 | <.001 | 0.010 | 1.008, 1.012 | 1.010 | <.001 |
| 2010-2019 | 0.107 | 1.097, 1.130 | 1.113 | <.001 | 0.097 | 1.077, 1.127 | 1.102 | <.001 |
| 2020-2022 | 0.039 | 0.963, 1.123 | 1.040 | .320 | -0.039 | 0.881, 1.050 | 0.962 | .385 |
| Sine | -0.094 | 0.853, 0.971 | 0.910 | .004 | -0.091 | 0.858, 0.971 | 0.913 | .004 |
| Cosine | 0.160 | 1.071, 1.285 | 1.173 | <.001 | 0.162 | 1.077, 1.285 | 1.176 | <.001 |
| Median Age | — | — | — | — | -0.017 | 0.909, 1.064 | 0.984 | .680 |
| Percent Male | — | — | — | — | 0.102 | 1.038, 1.182 | 1.107 | .002 |
| Unemployment Rate | — | — | — | — | -0.034 | 0.937, 0.997 | 0.967 | .033 |
| Alpha | 4.989 | 4.091, 5.887 | — | <.001 | 5.016 | 4.110, 5.922 | — | <.001 |
| Lag 1 | 0.103 | 0.078, 0.129 | — | <.001 | 0.100 | 0.075, 0.126 | — | <.001 |
| Lag 2 | 0.033 | 0.007, 0.060 | — | .013 | 0.029 | 0.003, 0.054 | — | .028 |
| Lag 3 | 0.038 | 0.011, 0.065 | — | .005 | 0.033 | 0.006, 0.059 | — | .015 |
| Lag 4 | 0.027 | 0.000, 0.053 | — | .049 | — | — | — | — |
| Lag 5 | 0.028 | 0.000, 0.055 | — | .046 | 0.022 | -0.004, 0.049 | — | .102 |
| Lag 6 | 0.041 | 0.014, 0.067 | — | .003 | 0.036 | 0.010, 0.063 | — | .007 |
| Lag 7 | 0.079 | 0.052, 0.105 | — | <.001 | 0.075 | 0.048, 0.101 | — | <.001 |
| Lag 8 | 0.051 | 0.024, 0.078 | — | <.001 | 0.048 | 0.021, 0.075 | — | <.001 |
| Lag 9 | 0.013 | -0.014, 0.040 | — | .330 | — | — | — | — |
| Lag 11 | 0.003 | -0.024, 0.030 | — | .824 | — | — | — | — |
| Lag 12 | 0.012 | -0.016, 0.040 | — | .389 | — | — | — | — |
| Lag 13 | 0.022 | -0.006, 0.049 | — | .118 | 0.020 | -0.007, 0.047 | — | .155 |
| Lag 14 | 0.072 | 0.047, 0.096 | — | <.001 | 0.068 | 0.043, 0.093 | — | <.001 |
| **Note**. b = unstandardized coefficient, IRR = incidence rate ratio. 95% CI = 95 percent confidence interval. Em-dashes indicate that a covariate was not included in the model, a moving average lag was not estimated, or that an incidence rate ratio is not interpretable (as is the case for the negative binomial dispersion parameter and moving average lag coefficients). Regression coefficients are reported to 3 decimal places to facilitate interpretation. | | | | | | | | |
| ^a^ The coefficient for daily apparent maximum temperature is expressed per-1°Fahrenheit. The corresponding per-1°Celsius incidence rate ratio is obtained by raising the IRR to the power of 1.8; the confidence interval limits transform identically and p-values are unchanged. Both unadjusted and adjusted models include linear terms representing pre-pandemic years (2010-2019) and post-pandemic years (2020-2022) as well as sine and cosine transformations of day-of-year to model annual cyclical patterns in violence incidence. Moving average terms were estimated at lags 1, 2, 3, 4, 5, 6, 7, 8, 9, 11, 12, 13, and 14 in the unadjusted model, and at lags 1, 2, 3, 5, 6, 7, 8, 13, and 14 in the adjusted model. | | | | | | | | |
| ^b^ Adjusted models include annual median age, percentage of males, and unemployment rate. | | | | | | | | |

# Table S3. Full Univariate GLARMA Models Examining the Relationship Between Daily Firearm Violence and Daily Deviation from Normal Temperature.

| **Variable ^a^** | **Unadjusted** | | | | **Adjusted ^b^** | | | |
| --- | --- | --- | --- | --- | --- | --- | --- | --- |
|  | b | 95% CI | IRR | p | b | 95% CI | IRR | p |
| Daily Deviation from Normal Temperature | 0.018 | 1.015, 1.022 | 1.018 | <.001 | 0.018 | 1.015, 1.022 | 1.019 | <.001 |
| 2010-2019 | 0.108 | 1.098, 1.130 | 1.114 | <.001 | 0.098 | 1.079, 1.128 | 1.103 | <.001 |
| 2020-2022 | 0.035 | 0.961, 1.116 | 1.035 | .360 | -0.046 | 0.875, 1.042 | 0.955 | .301 |
| Sine | -0.214 | 0.761, 0.856 | 0.807 | <.001 | -0.213 | 0.764, 0.855 | 0.808 | <.001 |
| Cosine | -0.168 | 0.798, 0.896 | 0.846 | <.001 | -0.168 | 0.799, 0.894 | 0.845 | <.001 |
| Median Age | — | — | — | — | -0.026 | 0.901, 1.054 | 0.974 | .512 |
| Percent Male | — | — | — | — | 0.106 | 1.042, 1.186 | 1.112 | .001 |
| Unemployment Rate | — | — | — | — | -0.038 | 0.934, 0.993 | 0.963 | .017 |
| Alpha | 5.110 | 4.177, 6.043 | — | <.001 | 5.154 | 4.209, 6.099 | — | <.001 |
| Lag 1 | 0.104 | 0.079, 0.130 | — | <.001 | 0.102 | 0.076, 0.127 | — | <.001 |
| Lag 2 | 0.031 | 0.004, 0.057 | — | .022 | 0.027 | 0.001, 0.052 | — | .039 |
| Lag 3 | 0.036 | 0.010, 0.062 | — | .008 | 0.030 | 0.004, 0.056 | — | .024 |
| Lag 4 | 0.027 | 0.001, 0.053 | — | .044 | — | — | — | — |
| Lag 5 | 0.028 | 0.002, 0.055 | — | .035 | 0.025 | -0.002, 0.052 | — | .065 |
| Lag 6 | 0.040 | 0.014, 0.066 | — | .003 | 0.036 | 0.010, 0.062 | — | .007 |
| Lag 7 | 0.082 | 0.056, 0.108 | — | <.001 | 0.079 | 0.053, 0.105 | — | <.001 |
| Lag 8 | 0.047 | 0.021, 0.074 | — | <.001 | 0.045 | 0.019, 0.072 | — | <.001 |
| Lag 13 | 0.024 | -0.002, 0.051 | — | .074 | 0.022 | -0.004, 0.049 | — | .102 |
| Lag 14 | 0.073 | 0.048, 0.097 | — | <.001 | 0.070 | 0.045, 0.094 | — | <.001 |
| **Note**. b = unstandardized coefficient, IRR = incidence rate ratio. 95% CI = 95 percent confidence interval. Em-dashes indicate that a covariate was not included in the model, a moving average lag was not estimated, or that an incidence rate ratio is not interpretable (as is the case for the negative binomial dispersion parameter and moving average lag coefficients). Regression coefficients are reported to 3 decimal places to facilitate interpretation. | | | | | | | | |
| ^a^ The coefficient for daily deviation from normal temperature is expressed per-1°Fahrenheit. The corresponding per-1°Celsius incidence rate ratio is obtained by raising the IRR to the power of 1.8; the confidence interval limits transform identically and p-values are unchanged. Both unadjusted and adjusted models include linear terms representing pre-pandemic years (2010-2019) and post-pandemic years (2020-2022) as well as sine and cosine transformations of day-of-year to model annual cyclical patterns in violence incidence. Moving average terms were estimated at lags 1, 2, 3, 4, 5, 6, 7, 8, 13, and 14 in the unadjusted model, and at lags 1, 2, 3, 5, 6, 7, 8, 13, and 14 in the adjusted model. | | | | | | | | |
| ^b^ Adjusted models include annual median age, percentage of males, and unemployment rate. | | | | | | | | |

# Table S4. Full Univariate GLARMA Models Examining the Relationship Between Daily Firearm Violence and Consecutive Days of High Heat.

| **Variable ^a^** | **Unadjusted** | | | | **Adjusted ^b^** | | | |
| --- | --- | --- | --- | --- | --- | --- | --- | --- |
|  | b | 95% CI | IRR | p | b | 95% CI | IRR | p |
| Consecutive Days of High Heat | 0.066 | 1.001, 1.139 | 1.068 | .045 | 0.065 | 1.000, 1.138 | 1.067 | .049 |
| 2010-2019 | 0.107 | 1.096, 1.130 | 1.113 | <.001 | 0.094 | 1.072, 1.125 | 1.098 | <.001 |
| 2020-2022 | 0.050 | 0.971, 1.139 | 1.052 | .214 | -0.036 | 0.879, 1.059 | 0.965 | .453 |
| Sine | -0.221 | 0.753, 0.853 | 0.801 | <.001 | -0.220 | 0.756, 0.852 | 0.802 | <.001 |
| Cosine | -0.163 | 0.798, 0.904 | 0.849 | <.001 | -0.163 | 0.800, 0.903 | 0.850 | <.001 |
| Median Age | — | — | — | — | -0.001 | 0.919, 1.085 | 0.999 | .976 |
| Percent Male | — | — | — | — | 0.117 | 1.050, 1.205 | 1.125 | <.001 |
| Unemployment Rate | — | — | — | — | -0.031 | 0.938, 1.002 | 0.970 | .065 |
| Alpha | 4.697 | 3.886, 5.508 | — | <.001 | 4.740 | 3.917, 5.563 | — | <.001 |
| Lag 1 | 0.117 | 0.092, 0.142 | — | <.001 | 0.114 | 0.089, 0.140 | — | <.001 |
| Lag 2 | 0.041 | 0.015, 0.068 | — | .002 | 0.038 | 0.011, 0.064 | — | .005 |
| Lag 3 | 0.048 | 0.021, 0.074 | — | <.001 | 0.045 | 0.018, 0.071 | — | .001 |
| Lag 4 | 0.038 | 0.012, 0.065 | — | .005 | 0.035 | 0.009, 0.062 | — | .009 |
| Lag 5 | 0.036 | 0.010, 0.063 | — | .008 | 0.033 | 0.007, 0.060 | — | .014 |
| Lag 6 | 0.040 | 0.013, 0.066 | — | .003 | 0.037 | 0.011, 0.063 | — | .005 |
| Lag 7 | 0.080 | 0.053, 0.106 | — | <.001 | 0.076 | 0.050, 0.103 | — | <.001 |
| Lag 8 | 0.049 | 0.022, 0.076 | — | <.001 | 0.044 | 0.017, 0.070 | — | .001 |
| Lag 9 | 0.022 | -0.004, 0.049 | — | .098 | — | — | — | — |
| Lag 13 | 0.027 | 0.001, 0.054 | — | .046 | 0.026 | -0.001, 0.053 | — | .057 |
| Lag 14 | 0.070 | 0.046, 0.095 | — | <.001 | 0.068 | 0.043, 0.092 | — | <.001 |
| **Note**. b = unstandardized coefficient, IRR = incidence rate ratio. 95% CI = 95 percent confidence interval. Em-dashes indicate that a covariate was not included in the model, a moving average lag was not estimated, or that an incidence rate ratio is not interpretable (as is the case for the negative binomial dispersion parameter and moving average lag coefficients). Regression coefficients are reported to 3 decimal places to facilitate interpretation. | | | | | | | | |
| ^a^ Both unadjusted and adjusted models include linear terms representing pre-pandemic years (2010-2019) and post-pandemic years (2020-2022) as well as sine and cosine transformations of day-of-year to model annual cyclical patterns in violence incidence. Moving average terms were estimated at lags 1, 2, 3, 4, 5, 6, 7, 8, 9, 13, and 14 in the unadjusted model, and at lags 1, 2, 3, 4, 5, 6, 7, 8, 13, and 14 in the adjusted model. | | | | | | | | |
| ^b^ Adjusted models include annual median age, percentage of males, and unemployment rate. | | | | | | | | |

# Table S5. Full Multivariate GLARMA Models Examining the Relationships Between Daily Firearm Violence and the Temperature Metrics.

| **Variable ^a^** | **Unadjusted** | | | | **Adjusted ^b^** | | | |
| --- | --- | --- | --- | --- | --- | --- | --- | --- |
|  | **b** | **95% CI** | **IRR** | **p** | **b** | **95% CI** | **IRR** | **p** |
| **Model 1: Daily Maximum Temperature** | | | | | | | | |
| Daily Maximum Temperature | 0.011 | 1.003, 1.018 | 1.011 | .004 | 0.010 | 1.003, 1.018 | 1.010 | .006 |
| Daily Deviation from Normal Temperature | 0.007 | 0.998, 1.016 | 1.007 | .126 | 0.007 | 0.999, 1.016 | 1.007 | .100 |
| Consecutive Days of High Heat | -0.001 | 0.936, 1.066 | 0.999 | .967 | -0.003 | 0.935, 1.064 | 0.997 | .929 |
| 2010-2019 | 0.108 | 1.098, 1.130 | 1.114 | <.001 | 0.098 | 1.078, 1.128 | 1.103 | <.001 |
| 2020-2022 | 0.032 | 0.958, 1.113 | 1.033 | .402 | -0.046 | 0.875, 1.042 | 0.955 | .298 |
| Sine | -0.123 | 0.813, 0.962 | 0.885 | .004 | -0.124 | 0.813, 0.959 | 0.883 | .003 |
| Cosine | 0.086 | 0.905, 1.313 | 1.090 | .362 | 0.077 | 0.898, 1.300 | 1.081 | .412 |
| Median Age | — | — | — | — | -0.022 | 0.905, 1.059 | 0.979 | .592 |
| Percent Male | — | — | — | — | 0.103 | 1.039, 1.183 | 1.109 | .002 |
| Unemployment Rate | — | — | — | — | -0.035 | 0.936, 0.996 | 0.965 | .025 |
| Alpha | 5.096 | 4.165, 6.027 | — | <.001 | 5.140 | 4.195, 6.085 | — | <.001 |
| Lag 1 | 0.102 | 0.076, 0.127 | — | <.001 | 0.099 | 0.074, 0.125 | — | <.001 |
| Lag 2 | 0.031 | 0.004, 0.057 | — | .023 | 0.027 | 0.001, 0.053 | — | .040 |
| Lag 3 | 0.037 | 0.010, 0.063 | — | .007 | 0.031 | 0.005, 0.058 | — | .020 |
| Lag 4 | 0.024 | -0.003, 0.050 | — | .077 | — | — | — | — |
| Lag 5 | 0.025 | -0.001, 0.052 | — | .064 | 0.022 | -0.005, 0.049 | — | .104 |
| Lag 6 | 0.040 | 0.014, 0.066 | — | .002 | 0.037 | 0.011, 0.063 | — | .005 |
| Lag 7 | 0.079 | 0.053, 0.105 | — | <.001 | 0.076 | 0.050, 0.102 | — | <.001 |
| Lag 8 | 0.049 | 0.023, 0.076 | — | <.001 | 0.048 | 0.021, 0.074 | — | <.001 |
| Lag 13 | 0.022 | -0.005, 0.049 | — | .110 | 0.021 | -0.006, 0.048 | — | .135 |
| Lag 14 | 0.071 | 0.047, 0.096 | — | <.001 | 0.068 | 0.044, 0.093 | — | <.001 |
| **Model 2: Daily Apparent Maximum Temperature** | | | | | | | | |
| Daily Apparent Maximum Temperature | 0.003 | 0.998, 1.008 | 1.003 | .181 | 0.003 | 0.998, 1.008 | 1.003 | .238 |
| Daily Deviation from Normal Temperature | 0.014 | 1.006, 1.022 | 1.014 | <.001 | 0.014 | 1.006, 1.023 | 1.014 | <.001 |
| Consecutive Days of High Heat | -0.010 | 0.927, 1.059 | 0.991 | .780 | -0.010 | 0.927, 1.059 | 0.990 | .777 |
| 2010-2019 | 0.108 | 1.099, 1.130 | 1.114 | <.001 | 0.098 | 1.079, 1.128 | 1.103 | <.001 |
| 2020-2022 | 0.031 | 0.959, 1.109 | 1.031 | .410 | -0.048 | 0.873, 1.041 | 0.953 | .285 |
| Sine | -0.172 | 0.777, 0.911 | 0.842 | <.001 | -0.176 | 0.775, 0.907 | 0.839 | <.001 |
| Cosine | -0.064 | 0.795, 1.106 | 0.938 | .446 | -0.078 | 0.785, 1.091 | 0.925 | .356 |
| Median Age | — | — | — | — | -0.024 | 0.903, 1.056 | 0.977 | .554 |
| Percent Male | — | — | — | — | 0.104 | 1.040, 1.184 | 1.110 | .002 |
| Unemployment Rate | — | — | — | — | -0.037 | 0.934, 0.994 | 0.964 | .020 |
| Alpha | 5.026 | 4.117, 5.935 | — | <.001 | 5.089 | 4.160, 6.018 | — | <.001 |
| Lag 1 | 0.102 | 0.077, 0.128 | — | <.001 | 0.101 | 0.075, 0.126 | — | <.001 |
| Lag 2 | 0.029 | 0.003, 0.055 | — | .026 | 0.026 | 0.001, 0.052 | — | .044 |
| Lag 3 | 0.033 | 0.006, 0.059 | — | .015 | 0.030 | 0.004, 0.057 | — | .023 |
| Lag 5 | 0.023 | -0.004, 0.050 | — | .092 | 0.022 | -0.005, 0.049 | — | .110 |
| Lag 6 | 0.039 | 0.013, 0.065 | — | .003 | 0.037 | 0.011, 0.063 | — | .005 |
| Lag 7 | 0.077 | 0.051, 0.104 | — | <.001 | 0.075 | 0.049, 0.102 | — | <.001 |
| Lag 8 | 0.050 | 0.023, 0.076 | — | <.001 | 0.047 | 0.021, 0.074 | — | <.001 |
| Lag 13 | 0.023 | -0.004, 0.050 | — | .098 | 0.022 | -0.006, 0.049 | — | .121 |
| Lag 14 | 0.071 | 0.046, 0.095 | — | <.001 | 0.069 | 0.044, 0.093 | — | <.001 |
| Note. b = unstandardized coefficient, IRR = incidence rate ratio. 95% CI = 95 percent confidence interval. Em-dashes indicate that a covariate was not included in the model, a moving average lag was not estimated, or that an incidence rate ratio is not interpretable (as is the case for the negative binomial dispersion parameter and moving average lag coefficients). Regression coefficients are reported to 3 decimal places to facilitate interpretation. | | | | | | | | |
| ^a^ Coefficients for temperature metrics measured in degrees (daily maximum, daily apparent maximum, and daily deviation from normal) are expressed per-1°Fahrenheit. Corresponding per-1°Celsius incidence rate ratios are obtained by raising each IRR to the power of 1.8; confidence interval limits transform identically and p-values are unchanged. Consecutive days of high heat is measured in days and is not rescaled. Both unadjusted and adjusted models include linear terms representing pre-pandemic years (2010-2019) and post-pandemic years (2020-2022) as well as a sine and cosine transformations of day-of-year to model annual cyclical patterns in violence incidence. Moving average terms were estimated at lags 1, 2, 3, 4, 5, 6, 7, 8, 13, and 14 in the unadjusted Model 1, and at lags 1, 2, 3, 5, 6, 7, 8, 13, and 14 in the adjusted Model 1 and both the unadjusted and adjusted Model 2. | | | | | | | | |
| ^b^ Adjusted models include annual median age, percentage of males, and unemployment rate. | | | | | | | | |

# Table S6. Full Univariate GLARMA Models Examining Whether a Non-Linear Relationship Exists Between Daily Firearm Violence and Consecutive Days of High Heat.

| **Variable ^a^** | **Unadjusted** | | | | **Adjusted ^n^** | | | |
| --- | --- | --- | --- | --- | --- | --- | --- | --- |
|  | **b** | **95% CI** | **IRR** | **p** | **b** | **95% CI** | **IRR** | **p** |
| Consecutive Days of High Heat | 0.151 | 1.012, 1.337 | 1.163 | .034 | 0.149 | 1.010, 1.333 | 1.160 | .036 |
| Consecutive Days of High Heat² | -0.023 | 0.944, 1.011 | 0.977 | .189 | -0.023 | 0.945, 1.011 | 0.977 | .188 |
| 2010-2019 | 0.108 | 1.099, 1.130 | 1.114 | <.001 | 0.094 | 1.073, 1.125 | 1.099 | <.001 |
| 2020-2022 | 0.044 | 0.967, 1.130 | 1.045 | .265 | -0.041 | 0.874, 1.054 | 0.960 | .388 |
| Sine | -0.216 | 0.758, 0.856 | 0.806 | <.001 | -0.215 | 0.759, 0.856 | 0.806 | <.001 |
| Cosine | -0.162 | 0.800, 0.905 | 0.850 | <.001 | -0.163 | 0.799, 0.903 | 0.850 | <.001 |
| Median Age | — | — | — | — | -0.001 | 0.919, 1.086 | 0.999 | .976 |
| Percent Male | — | — | — | — | 0.117 | 1.050, 1.204 | 1.124 | <.001 |
| Unemployment Rate | — | — | — | — | -0.031 | 0.938, 1.002 | 0.969 | .062 |
| Alpha | 4.628 | 3.832, 5.424 | — | <.001 | 4.689 | 3.876, 5.502 | — | <.001 |
| Lag 1 | 0.116 | 0.090, 0.141 | — | <.001 | 0.114 | 0.088, 0.139 | — | <.001 |
| Lag 2 | 0.041 | 0.014, 0.067 | — | .003 | 0.038 | 0.011, 0.064 | — | .005 |
| Lag 3 | 0.048 | 0.021, 0.075 | — | <.001 | 0.046 | 0.019, 0.072 | — | <.001 |
| Lag 4 | 0.036 | 0.010, 0.063 | — | .008 | 0.034 | 0.007, 0.060 | — | .013 |
| Lag 5 | 0.032 | 0.006, 0.059 | — | .018 | 0.031 | 0.004, 0.058 | — | .023 |
| Lag 6 | 0.040 | 0.013, 0.066 | — | .003 | 0.037 | 0.011, 0.064 | — | .005 |
| Lag 7 | 0.075 | 0.048, 0.101 | — | <.001 | 0.073 | 0.046, 0.099 | — | <.001 |
| Lag 8 | 0.048 | 0.021, 0.074 | — | <.001 | 0.046 | 0.019, 0.072 | — | <.001 |
| Lag 13 | 0.026 | -0.002, 0.053 | — | .065 | 0.024 | -0.003, 0.052 | — | .084 |
| Lag 14 | 0.070 | 0.045, 0.095 | — | <.001 | 0.068 | 0.043, 0.092 | — | <.001 |
| **Note**. b = unstandardized coefficient, IRR = incidence rate ratio. 95% CI = 95 percent confidence interval. Em-dashes indicate that a covariate was not included in the model, a moving average lag was not estimated, or that an incidence rate ratio is not interpretable (as is the case for the negative binomial dispersion parameter and moving average lag coefficients). Regression coefficients are reported to 3 decimal places to facilitate interpretation. | | | | | | | | |
| ^a^ Both unadjusted and adjusted models include linear terms representing pre-pandemic years (2010-2019) and post-pandemic years (2020-2022) as well as a sine and cosine transformations of day-of-year to model annual cyclical patterns in violence incidence. Moving average terms were estimated at lags 1, 2, 3, 4, 5, 6, 7, 8, 13, and 14 in both the unadjusted and adjusted models. | | | | | | | | |
| ^b^ Adjusted models include annual median age, percentage of males, and unemployment rate. | | | | | | | | |

# Table S7. Full Multivariate GLARMA Models Examining Whether a Non-Linear Relationship Exists Between Daily Firearm Violence and Consecutive Days of High Heat.

| **Variable ^a^** | **Unadjusted** | | | | **Adjusted ^b^** | | | |
| --- | --- | --- | --- | --- | --- | --- | --- | --- |
|  | b | 95% CI | IRR | p | b | 95% CI | IRR | p |
| Daily Maximum Temperature | 0.011 | 1.004, 1.018 | 1.011 | .004 | 0.010 | 1.003, 1.018 | 1.010 | .006 |
| Daily Deviation from Normal Temperature | 0.007 | 0.998, 1.016 | 1.007 | .125 | 0.007 | 0.999, 1.016 | 1.007 | .100 |
| Consecutive Days of High Heat | 0.003 | 0.873, 1.152 | 1.003 | .968 | 0.003 | 0.873, 1.152 | 1.003 | .969 |
| Consecutive Days of High Heat² | -0.001 | 0.966, 1.033 | 0.999 | .942 | -0.002 | 0.966, 1.032 | 0.998 | .928 |
| 2010-2019 | 0.108 | 1.099, 1.130 | 1.114 | <.001 | 0.098 | 1.078, 1.128 | 1.103 | <.001 |
| 2020-2022 | 0.031 | 0.959, 1.109 | 1.031 | .410 | -0.046 | 0.875, 1.042 | 0.955 | .298 |
| Sine | -0.121 | 0.816, 0.963 | 0.886 | .004 | -0.124 | 0.813, 0.959 | 0.883 | .003 |
| Cosine | 0.091 | 0.910, 1.318 | 1.095 | .336 | 0.077 | 0.898, 1.300 | 1.080 | .412 |
| Median Age | — | — | — | — | -0.022 | 0.905, 1.059 | 0.979 | .591 |
| Percent Male | — | — | — | — | 0.103 | 1.039, 1.183 | 1.109 | .002 |
| Unemployment Rate | — | — | — | — | -0.035 | 0.936, 0.996 | 0.965 | .025 |
| Alpha | 5.077 | 4.152, 6.002 | — | <.001 | 5.140 | 4.195, 6.085 | — | <.001 |
| Lag 1 | 0.101 | 0.075, 0.126 | — | <.001 | 0.099 | 0.074, 0.125 | — | <.001 |
| Lag 2 | 0.030 | 0.004, 0.055 | — | .023 | 0.027 | 0.001, 0.053 | — | .040 |
| Lag 3 | 0.033 | 0.007, 0.060 | — | .013 | 0.031 | 0.005, 0.058 | — | .020 |
| Lag 5 | 0.023 | -0.003, 0.050 | — | .087 | 0.022 | -0.005, 0.049 | — | .103 |
| Lag 6 | 0.039 | 0.013, 0.065 | — | .003 | 0.037 | 0.011, 0.063 | — | .005 |
| Lag 7 | 0.078 | 0.051, 0.104 | — | <.001 | 0.076 | 0.050, 0.102 | — | <.001 |
| Lag 8 | 0.050 | 0.023, 0.077 | — | <.001 | 0.048 | 0.021, 0.074 | — | <.001 |
| Lag 13 | 0.022 | -0.005, 0.049 | — | .110 | 0.021 | -0.007, 0.048 | — | .137 |
| Lag 14 | 0.070 | 0.046, 0.095 | — | <.001 | 0.068 | 0.044, 0.093 | — | <.001 |
| Note. b = unstandardized coefficient, IRR = incidence rate ratio. 95% CI = 95 percent confidence interval. Em-dashes indicate that a covariate was not included in the model, a moving average lag was not estimated, or that an incidence rate ratio is not interpretable (as is the case for the negative binomial dispersion parameter and moving average lag coefficients). Regression coefficients are reported to 3 decimal places to facilitate interpretation. | | | | | | | | |
| ^a^ Coefficients for temperature metrics measured in degrees (daily maximum and daily deviation from normal) are expressed per-1°Fahrenheit. Corresponding per-1°Celsius incidence rate ratios are obtained by raising each IRR to the power of 1.8; confidence interval limits transform identically and p-values are unchanged. Consecutive days of high heat is measured in days and is not rescaled. Both unadjusted and adjusted models include linear terms representing pre-pandemic years (2010-2019) and post-pandemic years (2020-2022) as well as a sine and cosine transformations of day-of-year to model annual cyclical patterns in violence incidence. Moving average terms were estimated at lags 1, 2, 3, 5, 6, 7, 8, 13, and 14 in both the unadjusted and adjusted models. | | | | | | | | |
| ^b^ Adjusted models include annual median age, percentage of males, and unemployment rate. | | | | | | | | |

# Table S8. Full Multivariate GLARMA Models Whether an Interaction Exists Between Daily Maximum Temperature and Consecutive Days of High Heat on Daily Firearm Violence.

| **Variable ^a^** | **Unadjusted** | | | | **Adjusted ^b^** | | | |
| --- | --- | --- | --- | --- | --- | --- | --- | --- |
|  | b | 95% CI | IRR | p | b | 95% CI | IRR | p |
| Daily Maximum Temperature | 0.011 | 1.004, 1.018 | 1.011 | .004 | 0.010 | 1.003, 1.018 | 1.010 | .006 |
| Daily Deviation from Normal Temperature | 0.007 | 0.998, 1.016 | 1.007 | .126 | 0.007 | 0.999, 1.016 | 1.007 | .101 |
| Consecutive Days of High Heat | 1.229 | 0.235, 49.686 | 3.418 | .368 | 1.150 | 0.220, 45.310 | 3.158 | .397 |
| Consecutive Days of High Heat² | 0.008 | 0.969, 1.048 | 1.008 | .690 | 0.007 | 0.969, 1.047 | 1.007 | .724 |
| Daily Maximum Temperature x Consecutive Days of High Heat | -0.014 | 0.958, 1.016 | 0.987 | .369 | -0.013 | 0.959, 1.017 | 0.987 | .398 |
| 2010-2019 | 0.108 | 1.099, 1.130 | 1.114 | <.001 | 0.098 | 1.079, 1.128 | 1.103 | <.001 |
| 2020-2022 | 0.031 | 0.959, 1.109 | 1.031 | .408 | -0.046 | 0.875, 1.042 | 0.955 | .303 |
| Sine | -0.120 | 0.816, 0.963 | 0.887 | .004 | -0.124 | 0.814, 0.960 | 0.884 | .003 |
| Cosine | 0.092 | 0.911, 1.320 | 1.096 | .330 | 0.078 | 0.899, 1.301 | 1.082 | .406 |
| Median Age | — | — | — | — | -0.021 | 0.905, 1.059 | 0.979 | .593 |
| Percent Male | — | — | — | — | 0.103 | 1.039, 1.183 | 1.108 | .002 |
| Unemployment Rate | — | — | — | — | -0.035 | 0.936, 0.996 | 0.965 | .026 |
| Alpha | 5.080 | 4.153, 6.007 | — | <.001 | 5.142 | 4.195, 6.089 | — | <.001 |
| Lag 1 | 0.101 | 0.075, 0.126 | — | <.001 | 0.099 | 0.074, 0.125 | — | <.001 |
| Lag 2 | 0.030 | 0.004, 0.055 | — | .023 | 0.027 | 0.001, 0.053 | — | .039 |
| Lag 3 | 0.034 | 0.007, 0.060 | — | .013 | 0.031 | 0.005, 0.058 | — | .019 |
| Lag 5 | 0.024 | -0.003, 0.051 | — | .083 | 0.023 | -0.004, 0.049 | — | .098 |
| Lag 6 | 0.039 | 0.013, 0.065 | — | .003 | 0.037 | 0.011, 0.063 | — | .005 |
| Lag 7 | 0.078 | 0.052, 0.104 | — | <.001 | 0.076 | 0.050, 0.102 | — | <.001 |
| Lag 8 | 0.050 | 0.023, 0.077 | — | <.001 | 0.048 | 0.021, 0.074 | — | <.001 |
| Lag 13 | 0.022 | -0.005, 0.049 | — | .117 | 0.020 | -0.007, 0.048 | — | .143 |
| Lag 14 | 0.071 | 0.046, 0.096 | — | <.001 | 0.069 | 0.044, 0.094 | — | <.001 |
| *Note.* b = unstandardized coefficient, IRR = incidence rate ratio. 95% CI = 95 percent confidence interval. Em-dashes indicate that a covariate was not included in the model, a moving average lag was not estimated, or that an incidence rate ratio is not interpretable (as is the case for the negative binomial dispersion parameter and moving average lag coefficients). Regression coefficients are reported to 3 decimal places to facilitate interpretation. | | | | | | | | |
| ^a^ Coefficients for temperature metrics measured in degrees (daily maximum and daily deviation from normal) are expressed per-a1°Fahrenheit. Corresponding per-1°Celsius incidence rate ratios are obtained by raising each IRR to the power of 1.8; confidence interval limits transform identically and p-values are unchanged. Consecutive days of high heat is measured in days and is not rescaled. The daily maximum temperature × consecutive days of high heat interaction converts in the same way. Both unadjusted and adjusted models include linear terms representing pre-pandemic years (2010-2019) and post-pandemic years (2020-2022) as well as a sine and cosine transformations of day-of-year to model annual cyclical patterns in violence incidence. Moving average terms were estimated at lags 1, 2, 3, 5, 6, 7, 8, 13, and 14 in both the unadjusted and adjusted models. | | | | | | | | |
| ^b^ Adjusted models include annual median age, percentage of males, and unemployment rate. | | | | | | | | |
